# Targeting Death Receptor 5 (DR5) for the imaging and treatment of primary bone and soft tissue tumors: an update of the literature

**DOI:** 10.3389/fmolb.2024.1384795

**Published:** 2024-09-02

**Authors:** Zakareya Gamie, Anja Krippner-Heidenreich, Craig Gerrand, Kenneth Samora Rankin

**Affiliations:** ^1^ Translational and Clinical Research Institute, Newcastle University, Newcastle upon Tyne, United Kingdom; ^2^ Princess Maxima Center of Pediatric Oncology, Utrecht, Netherlands; ^3^ Department of Orthopaedic Oncology, Royal National Orthopaedic Hospital NHS Trust, Stanmore, United Kingdom

**Keywords:** death, receptor, TRAIL, agonist, imaging, apoptosis, bone, sarcoma

## Abstract

**Background:**

Death Receptor 5 (DR5) is expressed on the surface of primary bone and soft tissue sarcoma cells, and its activation induces cell death primarily through apoptosis. The combination of DR5 agonists and commonly used chemotherapeutic agents, such as doxorubicin, can promote cell death. Currently, clinical trials are investigating the effectiveness of DR5 activation using new biological agents, such as bi-specific or tetravalent antibodies, in improving the survival of patients with relapsed or refractory cancers. Furthermore, investigations continue into the use of novel combination therapies to enhance DR5 response, for example, with inhibitor of apoptosis protein (IAP) antagonist agents [such as the second mitochondria-derived activator of caspase (SMAC) mimetics] and with immune checkpoint inhibitor anti-programmed death-ligand 1 (anti-PD-L1) or anti-programmed cell death-1 (anti-PD-1) antibodies. Other therapies include nanoparticle-mediated delivery of TRAIL plasmid DNA or TRAIL mRNA and stem cells as a vehicle for the targeted delivery of anti-cancer agents, such as TRAIL, to the tumor.

**Methods:**

Scoping review of the literature from November 2017 to March 2024, utilizing PubMed and Google Scholar.

**Results:**

New agents under investigation include nanoTRAIL, anti-Kv10.1, multimeric IgM, and humanized tetravalent antibodies. Developments have been made to test novel agents, and imaging has been used to detect DR5 in preclinical models and patients. The models include 3D spheroids, genetically modified mouse models, a novel jaw osteosarcoma model, and patient-derived xenograft (PDX) animal models. There are currently two ongoing clinical trials focusing on the activation of DR5, namely, IGM-8444 and INBRX-109, which have progressed to phase 2. Further modifications of TRAIL delivery with fusion to single-chain variable fragments (scFv-TRAIL), directed against tumor-associated antigens (TAAs), and in the use of stem cells focus on targeted TRAIL delivery to cancer cells using bi-functional strategies.

**Conclusion:**

*In vitro*, *in vivo*, and clinical trials, as well as advances in imaging and theranostics, indicate that targeting DR5 remains a valid strategy in the treatment of some relapsed and refractory cancers.

## Introduction

Connective tissue malignancies such as sarcoma are rare but can affect patients of all ages, have a variable but often poor prognosis, and have limited chemotherapeutic options (Bacon et al., 2023; Ducimetiere et al., 2011). They typically occur in the extremities but can also occur in a wide range of other anatomical locations, including the head and neck (e.g., maxillary and mandibular osteosarcoma). Patients, therefore, face esthetic and functional challenges that can significantly reduce their quality of life (Baumhoer et al., 2014; Cirstoiu et al., 2019). Bone and soft tissue sarcomas, in addition to some hematopoietic malignancies, such as multiple myeloma, are susceptible to TRAIL-induced apoptosis and therapy in combination with or without other chemotherapeutic options (Snajdauf et al., 2021; Subbiah et al., 2023; van der Horst et al., 2021; Trivedi and Mishra, 2015). The current understanding is that TRAIL binds to DR5/TRAILR2 more efficiently than it does to DR4/TRAILR1 through a stepwise binding mechanism (Kelley et al., 2005). However, many cancer cells are sensitive to apoptosis through both DR4 and DR5 activation (Pimentel et al., 2023a). Despite the lack of progression from phase 2 to phase 3 clinical trials of the early DR5 agonistic antibodies, TRAIL therapy remains a promising therapeutic approach, particularly as part of combination therapy (Montinaro and Walczak, 2023; Di Cristofano et al., 2023).

The mechanism of TRAIL-induced apoptosis, the transcriptional regulation, and modulation of the localization of the receptors (Min et al., 2019), and also TRAIL-mediated non-apoptotic signaling have been reviewed extensively (Gamie et al., 2017). There are promising TRAIL derivatives, such as the fusion of single-chain variable fragments (scFv-TRAIL), directed against a tumor-associated antigen (TAA) (Hutt et al., 2018). This allows for better target specificity and can be combined with other biological entities, such as immune checkpoint blockade, which is itself a potential treatment for bone and soft tissue sarcomas (Thanindratarn et al., 2019; Fazel et al., 2023). In mixed T-cell and cancer-cell culture experiments, the scFv-PD-L1:TRAIL derivative enhanced the cytotoxicity of TRAIL by exhibiting a multi-fold therapeutic effect, which includes reactivating T-cells and stimulating IFNγ production, thereby upregulating programmed death-ligand 1 (PD-L1) and sensitizing cancer cells to apoptosis by TRAIL (Hendriks et al., 2016). However, more recent experiments have shown that the knockdown or knockout of PD-L1 may sensitize certain cancer cells to TRAIL via a non-canonical mechanism (Pimentel et al., 2023b).

Improving the effectiveness of novel TRAIL agonists and derivatives and combining TRAIL with current chemotherapeutics require evidence of the protein expression of death receptors (DRs) and target antigens, immune-based stratification, and clinical trials to assess efficacy. The purpose of the current scoping review of the literature is, therefore, to provide a general overview and update on the effectiveness of therapies developed to activate DR5 for connective tissue malignancies such as sarcoma, modes of action, and models developed to test them, ongoing clinical trials, and effectiveness. A scoping review search strategy was used to assess a body of literature, clarify concepts, and identify areas for further investigation. This was performed using the following article subheadings, keywords, and index terms: Death; Receptor; TRAIL; Target; Imaging; Apoptosis; Bone; Sarcoma; Malignancy; Stem; Cell; *In vitro*; *In vivo*; Clinical; and Trial. One database (PubMed and Google Scholar) and one concept at a time were searched. Inclusion criteria were studies from 2017 to 2024; any *in vitro*, *in vivo*, or clinical studies in the English language investigating DR5 as a target for imaging or as an agonist to induce apoptosis in primary bone and soft tissue tumors; and published and unpublished work (gray literature), supplements, proceedings of meetings, or conference abstracts. There was an initial screening of titles and abstracts, followed by full manuscript reviews.

## DR5 structural features and signal transduction

DR5 receptors are expressed on the cell surface, and like other members of the TNF receptor superfamily, TRAIL receptors pre-oligomerize in the absence of a ligand via their pre-ligand-binding domain (PLAD) (Chan et al., 2000). The stoichiometry of TRAIL receptor homotypic oligomerization is not fully clarified yet; however, homodimers, homotrimers, and even heterodimers of the tumor necrosis factor receptor (TNFR), including DR4 and DR5, have been described (Chan et al., 2000; Neumann et al., 2014; Richter et al., 2012). The natural ligands of DR5 are homotrimeric membrane-bound TRAIL and soluble TRAIL, the shed form of membrane-bound TRAIL (Wang Y. et al., 2021). Receptor clustering is a precisely controlled process, and its triggering has been described to involve the formation of hexagonal patterns (Scott et al., 2009). The dimerization of DR5 trimers resulting in the hexagonal structure has been previously hypothesized by studies investigating DR5 clusters on the surface of apoptotic cells (Scott et al., 2009; Martin et al., 2005; Pan et al., 2019). More recently, it has been shown that the ligand-induced dimerization/trimerization of the transmembrane domain of DR5 and other members of the TNFR superfamily, such as OX40 and TNFR2, drive higher-order ligand/receptor oligomerization and downstream signaling (Pan et al., 2019).

**FIGURE 1:**
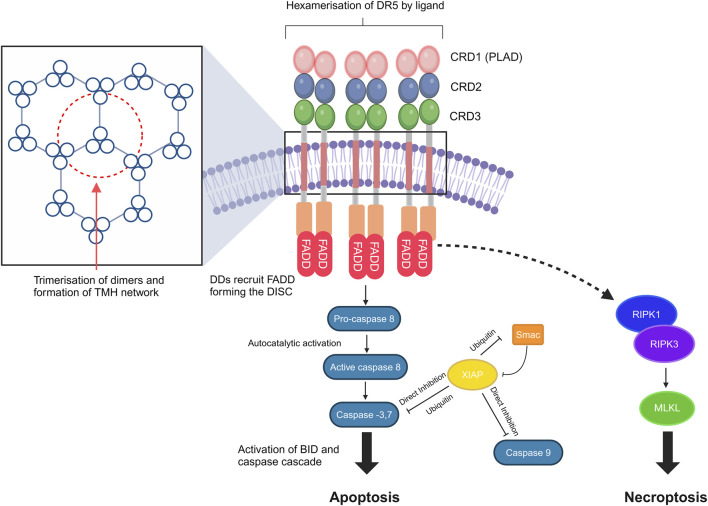
DR5 structure, clustering, and signal transduction. DR signaling leads to caspase-8 activation and cleavage of BID, resulting in mitochondrial outer-membrane permeabilization (MOMP). Transmembrane helix (THM) clustering induced by the targeted agents is required for DR5 signaling. Non-apoptotic pathways may also be activated, which result in the formation of secondary complexes and activation of necroptosis via the ripoptosome [core components are receptor-interacting serine/threonine-protein kinase 1 (RIPK1) and receptor-interacting serine/threonine-protein kinase 3 (RIPK3)]. DDs, death domains; FADD, Fas-associated death domain; DISC, death-inducing signaling complex; PLAD, pre-ligand association domain; BID, BH3-interacting-domain death agonist; XIAP, X-linked inhibitor of apoptosis protein; MLKL, mixed lineage kinase domain-like pseudokinase. The figure was created with BioRender (https://biorender.com/).

Unique to DRs, including TNFR1, DR3, DR4, DR5, DR6, and ectodysplasin A receptor (EDAR) and a nerve growth factor receptor (NGFR), are the names given to the intra-cellular death domain (DD) (Green, 2022; Horiuchi et al., 2010). DDs bind to different DD-containing adapter proteins in the next step of the signaling pathway, such as the Fas-associated death domain (FADD), forming the death-inducing signaling complex (DISC), which binds to and dimerizes caspase 8, thereby activating it and cleaving BID, giving rise to mitochondrial outer-membrane permeabilization (MOMP), resulting in apoptosis (Green, 2022). Non-apoptotic pathways can also be activated, resulting in the formation of secondary complexes and activation of necroptosis via the ripoptosome and protumoral, survival, motility, and inflammatory effects via NF-kB activation (Guerrache and Micheau, 2024) (Figure 1).

## DR5 expression and cancer-associated mutations of DR5

For a cell to be susceptible to apoptosis, at least one type of the two DD-containing TRAIL receptors must be expressed on the cell surface. DR4 and DR5 have been found in bone sarcomas (Ewing’s sarcoma, osteosarcoma, and chondrosarcoma) and hematopoietic tumors such as myeloma, with the DR5 receptor being the most frequently expressed (Subbiah et al., 2023; Picarda et al., 2010; Surget et al., 2012; Chen et al., 2013). Further investigations are needed to better understand the expression levels and somatic mutations found in DRs and investigate how they influence signaling. In non-small-cell lung and breast cancer, most of the mutations identified to date affect the intracellular domain of DR5 (Bin et al., 2007) or heterodimerization (Neumann et al., 2014). Allelic loss has been found on chromosomes 8p21–22, where DR5 is located, in head and neck squamous cell cancer (Fisher et al., 2001) and non-Hodgkin’s lymphoma (Lee et al., 2001). There may also be a dominant-negative effect where mutant DR5 can inhibit the activation of DR4 via competition for ligand binding (Bin et al., 2007). Other important mutations occur in the extracellular PLAD, which can prevent ligand binding by interfering with homotypic pre-ligand receptor oligomerization (Bin et al., 2007). Uncertainties exist in the knowledge of the prevalence and effects of these mutations in different cancer types, including bone and soft tissue tumors such as sarcomas; however, common similar mutations have been found repeatedly, resulting in an inability to properly recruit the FADD (Bin et al., 2007). A study investigating osteosarcoma tumor samples and cell lines revealed mutations mainly in the DR4 gene that affect both the ligand binding and death domain regions and may be implicated in osteosarcoma pathogenesis (Dechant et al., 2004).

## New biologics that target DR5

New drugs have been developed that target and achieve better activation of DR5 signaling in the last few years, following our previous review (Gamie et al., 2017). A challenge for TRAIL-based therapies is the ability to distribute and deliver TRAIL to the tumor, and combinations with nanoparticles (NPs) have also been developed to help enable this (Sadeghnezhad et al., 2019). The therapeutics are summarized in Table 1 and discussed in more detail below. Previous studies have utilized TRAIL agonists or recombinant TRAIL with mixed results in terms of progression-free survival and overall survival, which may be due to the inclusion of unselected patients (Gampa et al., 2023). Table 2 summarizes current ongoing clinical trials targeting DR4 and DR5 in orthopedic-related tumors.

**TABLE 1:** Novel DR5-targeting therapeutics developed.

DR5-activating construct	Structure/mode of action
Anti-Kv10.1 nanobody	Anti-Kv10.1 nanobody fused to single-chain TRAIL (scTRAIL). Dual-epitope targeting and enhanced hexamerization
DS-8273a	Humanized monoclonal agnostic antibody (super agonist) binding specifically to DR5 capable of receptor-enhanced multimerization
Zapadcine-1	Fully humanized antibody agonist of DR5 (zaptuzumab) linked to a toxic inhibitor of tubulin (MMAD) monomethyl auristatin D
INBRX-109	Humanized tetravalent antibody targeting DR5
ABBV-621(eftozanermin)	Hexavalent TRAIL-Fc fusion protein that activates both DR4 and DR5
IGM-8444 (aplitabart)	Multivalent IgM antibody agent targeting DR5
HexaBody-DR5/DR5	Two noncompeting DR5-specific immunoglobulin G1 (IgG1) antibodies, each carrying an E430G mutation in the Fc domain enhancing Fc–Fc interactions and hexamerization
Circularly permuted TRAIL (CPT)	A recombinant mutant of human TRAIL, which has a circularly permuted extracellular sequence of native TRAIL

**TABLE 2:** Clinical trials investigating DR-targeting therapeutics for primary bone tumors.

rTRAIL			DR4 targeting			DR5 targeting			DR5 targeting		
ABBV-621(eftozanermin)–hexavalent TRAIL-Fc fusion protein			Mapatumumab			INBRX-109			IGM-8444		
Cancer type	Clinical trial registration number	Phase	Cancer type	Clinical trial registration number	Phase	Cancer type	Clinical trial registration number	Phase	Cancer type	Clinical trial registration number	Phase
Advanced solid tumors and hematological malignancies	NCT03082209	Phase 1—completed	Multiple myeloma	NCT00315757	Phase 2 – completed	Solid tumors, malignant pleural mesothelioma, gastric, colorectal, sarcoma (Ewing and chondrosarcoma), and pancreatic cancer	NCT03715933	Phase 1—recruiting	Solid tumors, colorectal cancer, lymphoma (non-Hodgkin’s and small lymphocytic), sarcoma (chondrosarcoma), and leukemia (chronic lymphocytic and acute)	NCT04553692	Phase 1a/1b—recruiting
Multiple myeloma	NCT04570631 - With IV or subcutaneous (SC) bortezomib and oral dexamethasone	Phase 2 - active, not recruiting				Chondrosarcoma	NCT04950075	Phase 2—recruiting			

### Novel anti-Kv10.1 nanobody

Kv10.1 is a voltage-gated potassium channel overexpressed in cancer cells. It is considered to be a TAA, and, therefore, an anti-Kv10.1 nanobody has been produced and fused to a single-chain TRAIL (scTRAIL), named VHH-D9-scTRAIL, a development in the concept of scTNF and scTRAIL (Krippner-Heidenreich et al., 2008; Schneider et al., 2010). VHH-D9-scTRAIL has demonstrated enhanced apoptosis *in vitro* in human prostate cancer cells (DU-145), using live cell imaging and caspase assays (Hartung et al., 2020). VHH-D9-scTRAIL is based on the single-chain variant of TRAIL first described by Krippner-Heidenreich et al. (2008), with the advantage of including nanobodies to induce a strong and rapid apoptotic response in different tumor models, such as 2D culture and spheroids, involving a human prostate cancer cell line (DU-145) and a pancreatic cancer cell line (Caplan-1) and is more potent than the ScFv version. In the 3D culture model, the spheroids were monitored in the Incucyte system, and the change in size was determined by the degree of the surface occupied by green fluorescence, which was monitored as a measure of growth (Hartung et al., 2020).

### DR5 agonist, DS-8273a

DS-8273a is a new generation of DR5 super-agonists capable of receptor-enhanced multimerization. Four of these multivalent targeting agents are being investigated in clinical trials studying malignant solid tumors, including sarcomas, i.e., ABBV-621, GEN 1029, INBX-109, and BI 905711. A number of these are undergoing first-in-human studies (Wang B. T. et al., 2021). The first-in-human study of the monoclonal antibody DR5 agonist DS-8273a is ongoing in patients with advanced solid tumors. DS-8273a can induce apoptosis in myeloid-derived suppressor cells (MDSCs) *ex vivo* and reduce peripheral blood numbers to levels found in healthy volunteers. Treatment is inversely correlated with the length of progression-free survival (Dominguez et al., 2017). This agent has also been investigated in combination with nivolumab (an anti-PD-1 antibody) for treating unresectable stage 3 or 4 melanoma in phase 1 clinical trials, completed in March 2024 (ClinicalTrials.gov Identifier: NCT02983006).

### Zapadcine-1

Following on from the significant *in vitro* and *in vivo* efficacy of a humanized monoclonal antibody zaptuzumab in lung carcinoma, the same group demonstrated that a novel anti-DR5 antibody-drug conjugate, Zapadcine-1, possesses a high-potential therapeutic efficacy against leukemia and solid tumors (Zhang et al., 2019; Zheng et al., 2023). It is a fully-humanized agonist of DR5 (zaptuzumab) linked to a toxic inhibitor of tubulin, monomethyl auristatin D (MMAD). Zapadcine-1 was able to eliminate cancer cells in cell-derived xenografts and patient-derived xenograft (PDX) models of human lymphocytic leukemia in a dose-dependent manner and in a mouse model of lung cancer. Zapadcine-1 has an acceptable safety profile in rat and cynomolgus monkey models (Zhang et al., 2019; Zheng et al., 2023).

### INBRX-109

Another agent known as INBRX-109, a humanized tetravalent antibody targeting DR5, is being studied in a randomized, placebo-controlled phase 2 study in patients with unresectable and/or metastatic conventional chondrosarcoma (ClinicalTrials.gov Identifier: NCT04950075). Participants with radiological evidence of progression in the trial can be unblinded and offered crossover to the INBRX-109 agent if they have been receiving a placebo. The drug has been granted orphan drug designation. Trials were initially paused due to concerns about liver toxicity but were resumed to exclude high-risk patients. The primary endpoint is progression-free survival for over 3 years (Newton, 2023).

### ABBV-621 (eftozanermin)

ABBV-621 (eftozanermin) is an engineered second-generation TRAIL agonist containing IgG1-Fc linked to a single-chain trimer of TRAIL subunits that was tested in phase 1 clinical trials (ClinicalTrials.gov Identifier: NCT03082209) in patients with previously treated solid tumors or hematological malignancies. This hexavalent TRAIL-Fc fusion protein activates both DR4 and DR5 with nanomolar affinity, inducing cell death in the COLO-205 cell line at concentrations ranging from 1 to 10 nmol/L (Phillips et al., 2021). It has been well tolerated in the first-in-human study, achieving tumor regression in colorectal cancer (LoRusso et al., 2022). However, there was a requirement for the evaluation of the expression of DR4/DR5 in fresh biopsies and the clinical response to this agent. Regarding bone tumors, it has been investigated in cell line models that have included multiple myeloma, and in combination with bortezomib, it has resulted in greater anti-tumor activity (Smith, 2020). A clinical trial on relapsed/refractory multiple myeloma patients is planned (Smith, 2020). Investigations have also demonstrated effectiveness in solid tumor colorectal cancer PDX models (Phillips et al., 2021).

### IGM-8444 (aplitabart)

IGM-8444 (aplitabart) is an IgM antibody agent targeting DR5 and is a potent inducer of apoptosis, particularly when combined with a Bcl-2 inhibitor. This has been demonstrated in colorectal and lung cancer xenograft models and a gastric GXF251 PDX model without concern for hepatotoxicity (Wang B. T. et al., 2021). This agent has 10 binding sites to DR5, inducing effective multimerization (Wang B. T. et al., 2021). It can be administered i.v. and is being studied alone or in combination with relapsed, refractory, and newly diagnosed cancer patients in a randomized trial of 430 participants (ClinicalTrials.gov identifier: NCT04553692), which is still recruiting. This includes treatment in combination with birinapant [a second mitochondria-derived activator of caspase (SMAC) mimetic, which is an antagonist of the inhibitor of apoptosis (IAP) proteins] in sarcoma and venetoclax (a Bcl-2 inhibitor) in chondrosarcoma. Multimeric IGM-844 can also synergize with ABT199, a Bcl-2 inhibitor. It exhibited enhanced cytotoxicity and *in vivo,* efficacy in PDX tumor models, and no concern for increased hepatotoxicity in an investigation was noted using primary human hepatocytes. In addition, in cynomolgus monkeys, repeated IGM-844 dosing did not show a significant increase in liver enzymes compared to control (Wang B. T. et al., 2021).

### HexaBody-DR5/DR5

There has been a development in dual-epitope targeting and enhanced hexamerization by DR5 antibodies as a novel approach to induce potent antitumor activity through the DR5 agonist. HexaBody-DR5/DR5 (GEN1029) has demonstrated enhanced hexamerization through the binding of two different DR5 epitopes on the cell surface (van der Horst et al., 2021). These multimeric IgM antibodies have also demonstrated effectiveness through efficient receptor clustering in gastric, skin, and squamous cell carcinomas. It has demonstrated little or no human hepatocyte cytotoxicity (Sinclair, 2021). It has been shown to work synergistically with birinapant in multiple cancer cell lines *in vitro*, including HT1080, HCT116, and NSCLC cells, and in studies of colorectal, sarcoma, and head and neck cancers (Sinclair, 2021).

HexaBody-DR5/DR5 has undergone clinical trials in solid cancers, which have been open-labeled and multi-centered. HexaBody-DR5/DR5 was developed for relapsed or refractory multiple myeloma and can induce significant cytotoxicity in primary multiple myeloma cells. It has shown potent antitumor activity in a variety of PDX models (gastric, urothelial, and colorectal cancers) in a preclinical proof-of-concept study (Overdijk et al., 2019). Recently, however, the phase 1 and expansion phase 2a studies in solid tumors were terminated due to safety concerns for unspecified reasons (NCT03576131) (Di Cristofano et al., 2023; Carter and Rajpal, 2022).

### Circularly permuted TRAIL

Another therapeutic is circularly permuted TRAIL (CPT), a recombinant mutant of human TRAIL, which has a circularly permuted extracellular sequence of native TRAIL (Fang et al., 2005). It has better stability, a longer half-life, and less toxicity toward normal cells than wild-type TRAIL, and when combined with thalidomide and dexamethasone, it can prolong survival in relapsed/refractory multiple myeloma patients. It has progressed from phase 2 (Leng et al., 2017) to phase 3 clinical trials (Chen et al., 2021).

## Nanoparticle-mediated delivery of TRAIL

Nanoparticle-mediated delivery of TRAIL genetic material has been utilized as a form of TRAIL gene therapy, e.g., using PEG-grafted chitosan to deliver TRAIL plasmid DNA to glioblastoma tumor cells (Wang et al., 2015). The uptake of the TRAIL plasmid-loaded nanoparticles enables the expression and liberation of TRAIL in the tumor microenvironment, thereby inducing apoptosis (Wang et al., 2015). A publication this year also detailed how TRAIL mRNA can be delivered using lipid NPs. It consists of intratumoral injection in an *in vivo* COLO-205 tumor cell study, resulting in enhanced apoptosis and necrosis (da Silva et al., 2024). However, dosing and long-term effects require further investigation and studies in other cancer types, such as bone and soft tissue tumors.

## Measures to avoid hepatotoxicity

Hepatotoxicity has been an ongoing concern regarding the use of TRAIL-related therapeutics (Gores and Kaufmann, 2001; Papadopoulos et al., 2015). There have been certain measures implemented to help reduce hepatotoxicity; for example, when using TRAIL in combination with the proteasome inhibitor bortezomib, the use of a lower concentration of bortezomib-induced apoptosis in treatment-resistant cancer cell lines (hepatoma, pancreatic, and colon) but not in primary hepatocytes by working within a specific therapeutic window (Koschny et al., 2007; Yuan et al., 2018; Diaz and Haisma, 2021). Developments have also been made to DR5 agonists using IgM antibodies, such as the multivalent agonistic antibody IGM-8444, which has demonstrated reduced cytotoxicity in primary human hepatocytes *in vitro* (Carter and Rajpal, 2022) and in cynomolgus monkeys (Wang B. T. et al., 2021). HexaBody DR5/DR5 has also demonstrated reduced human hepatocyte cytotoxicity compared to COLO-205 cells in 24-h *in vitro* cytotoxicity assays. INBRX-109 was engineered to limit hepatotoxicity by limiting its valency from 6 (hexavalent) to 4 (tetravalent) (Di Cristofano et al., 2023). It has also demonstrated reduced hepatotoxicity in preclinical studies *in vitro* and xenograft models and demonstrated favorable safety profiles in patients with unresectable/metastatic chondrosarcoma (Subbiah et al., 2023).

## Animal model development

There has been a need to develop animal models to more accurately understand the progression of sarcoma and test new therapeutics. There have been cell line xenograft models, PDX models, and genetically modified mouse models produced (Yagolovich et al., 2020; Guijarro et al., 2014) with new developments, such as a dedifferentiated chondrosarcoma orthotopic mouse model (Pringle et al., 2022). In particular, for DR5 activation, a colon cancer xenograft model has been developed to assess the efficacy and pharmacokinetic profile of a DR5 ligand, a genetically modified DR5-B variant selective for DR5 (Gasparian et al., 2009), revealing dual pro-tumoral and antitumoral effects related to the concentration and mode of administration (Yagolovich et al., 2020).

For personalized therapy in sarcoma, a number of publications detailed the production of PDX models using surgical orthotopic implantation (biopsy or surgical specimen, which is frequently implanted subcutaneously) to mimic the clinical disease and be used to assess agents for treating drug-resistant osteosarcoma as monotherapy or combination therapy (Higuchi et al., 2021; Meohas et al., 2018). There have been developments in the creation of models for use in studying new therapeutics, mainly for the treatment of osteosarcoma. PDX models can be used to reproduce recurrent or recalcitrant disease and can help in studying experimental agents and drug combinations. More investigations are required using chondrosarcoma tissue to produce patient-derived chondrosarcoma models. Subcutaneous engraftment of patient tissue can fail, as reported. This is potentially due to the low aggressiveness of the tumor or a small sample size from the core biopsy (Meohas et al., 2018). This is in contrast to osteosarcoma and Ewing’s sarcoma, which appear to be faithful and stable preclinical models (Guijarro et al., 2014; Nanni et al., 2019).

Osteosarcoma of the mandible has a reported 5-year survival rate of 84% with adjuvant chemotherapy (Nissanka et al., 2007). Treatment methods include wide radical resection, adjuvant chemotherapy, and radiotherapy (Bertin et al., 2020). An animal model has been developed, which can, in the future, help investigate the effectiveness of novel agents for jaw osteosarcoma (Bertin et al., 2019). It, however, requires comparative studies with models using long bones to study differences in the bone microenvironment and behavior. The cell lines engrafted were K7M2, POS-1, and MOS-J from mouse osteosarcoma, primary patient, and C57BL/6J mouse osteosarcoma origins, respectively (Bertin et al., 2019). Osteolytic lesions can be induced using these cell lines similar to those in previous paratibial models (Bertin et al., 2019). PDX models can be challenging due to the requirement of a sufficient amount of fresh tissue, and it can be challenging to achieve successful engraftment. However, they may be valuable for personalized therapies and for the small number of patients with rare cancers who are eligible for experimental therapies (Fujii et al., 2020). The benefit of genetically engineered models of osteosarcoma is the appearance of the tumor in easily accessible sites, such as long bones, compared to other models of cancer, such as abdominal tumors. A challenge has been developing models of tumor heterogeneity, spontaneous osteosarcoma, and micrometastases and monitoring their development in these models (Fujii et al., 2020). Therefore, there has been growing interest in the development of *in vivo* imaging of tumor cells, which includes bioluminescence, micro-CT, and positron emission tomography (PET) scanning (de Jong et al., 2014).

## *In vitro* model system development

Disadvantages of animal models include cost, labor required, and prediction of drug safety (Van Norman, 2019). There has been increasing use of spheroids, organoids, and 3D cell cultures, terms used interchangeably, to try to replicate malignant tissues and microtumors (Bialkowska et al., 2020; Gunti et al., 2021). A number of methods have been described to generate them, such as the hanging drop method, which can exist as co-cultures with or without stromal cells and an extracellular matrix and better mimic tumor structure than 2D cell culture (Bialkowska et al., 2020; Gunti et al., 2021; Jensen and Teng, 2020). They have played a role in studying the chondrosarcoma microenvironment and factors such as hypoxia and pH, resistance to drug therapy, and the question of what the effect of 3D microenvironments is on susceptibility to apoptosis (Bialkowska et al., 2020). Stohr et al. (2020) found that there can be TRAIL-resistant subpopulations in spheroids generated by using HCT116 and NCI-H460 cells seeded in Terasaki multi-well plates and placed in humid chambers in the incubator (Stohr et al., 2020). They lacked DR4 and DR5 at the interface of proliferating and quiescent cells, which protect the TRAIL-sensitive cells residing in the inner spheroid layers. This highlights the importance of studying and comparing the results from 3D as well as 2D models. This study also found that the COX-2 inhibitor, celecoxib, could upregulate DR4 and DR5 through increased ER stress (Stohr et al., 2020).

## Imaging to detect DR5 cell surface levels and apoptosis induction

Imaging of solid tumors that relies on size-based criteria may be limited in assessing response; furthermore, bone scans for cancer may be limited in their sensitivity and specificity for treatment response (Cook and Goh, 2020). Imaging modalities such as PET and single-photon emission computed tomography (SPECT) can improve the diagnosis and treatment response. There have been tools developed to monitor the response to DR5 therapy, for example, *in vivo* by using fluorescence-labeled anti-DR5 with the reporter construct C-Luc-DEVD-N-Luc. Light is emitted upon the caspase-mediated cleavage of this construct and subsequent dimerization of C-Luc and N-Luc in the presence of externally supplied luciferin (Weber et al., 2014; Weber et al., 2013). In addition to DR5 being utilized as a potential imaging marker (Wang S. et al., 2021), PET has been used in combination with F18-duramycin to assess the response to DR5 agonists and early apoptosis in humans. Duramycin is a 19-amino acid peptide that can detect apoptosis by targeting and binding to phosphatidylethanolamine (PE) and phosphatidylserine (PS), which are expressed in the early stages of apoptosis (Cook and Goh, 2020; Li et al., 2019; Zhao et al., 2008). In preliminary data, using micro-PET and micro-CT scanning, the colorectal cancer cell line COLO-205 was injected into the shoulder of mice and found to be more drug-sensitive when using bioluminescence imaging (BLI), i.e., it was targeted more efficiently by duramycin, indicating greater apoptosis induction by the DR5 agonist agent AMG655 (Zhang et al., 2019; Kim et al., 2012).

The 177Lu or 89Zr radio-labeled anti-DR5 antibody CTB006 (89Zr-CTB006) PET/CT has been useful for screening cancers with DR5 overexpression, such as gastrointestinal cancer (Wang S. et al., 2021). Similar studies are required for bone and soft tissue tumors. Reduced DR5 expression may explain the poor response to DR5 agonists in clinical trials. Using this technology can help identify patients who may benefit from DR5 agonist therapy in a non-invasive manner. The inability to achieve the endpoint in previous trials may be due to the inability to accurately quantify the degree of DR5 expression in immunohistochemistry (IHC). DR5 expression was lower than that previously reported and requires further investigation. 89Zr-CTB006 PET/CT has also been able to detect DR5 levels in PDX mouse models with RNAscope scores of 3 and 4, which is a method to detect specific gene expression that is more sensitive and specific than IHC methods (Wang S. et al., 2021).

**FIGURE 2:**
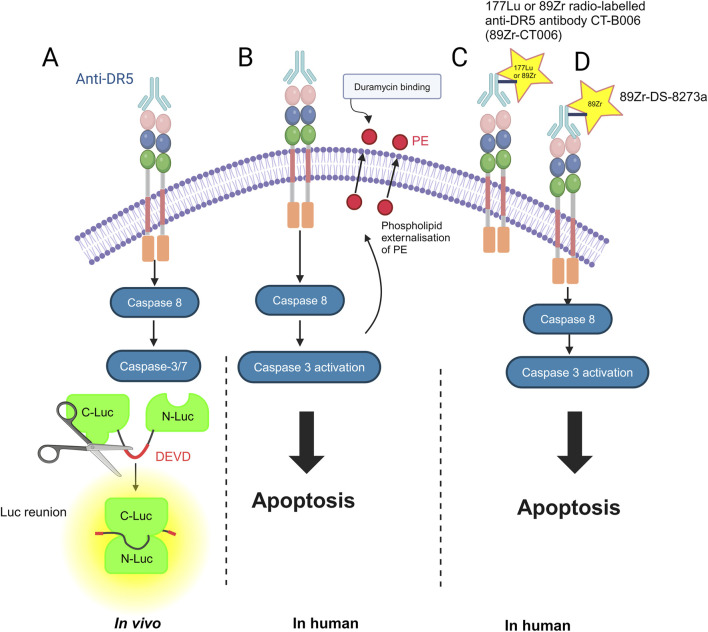
Methods for imaging DR5 and its cell death-inducing activity: **(A)** *in vivo* by using fluorescence-labeled anti-DR5 with the reporter construct C-Luc-DEVD-N-Luc. N-terminal (N-Luc) and C-terminal (C-Luc) (Weber et al., 2013). **(B)** In humans, duramycin, a 19-amino acid peptide that can detect apoptosis by targeting and binding to phosphatidylethanolamine (PE) (Li et al., 2019). **(C)** 177Lu or 89Zr radio-labeled anti-DR5 antibody CTB006 (89Zr-CTB006) (Wang S. et al., 2021). **(D)** 89Zr-DS-8273a is also being investigated as a theranostic for anti-DR5 cancer therapy (Burvenich et al., 2016). The figure was created with BioRender (https://biorender.com/).

Furthermore, 89Zr-DS-8273a is being investigated as a theranostic agent for anti-DR5 cancer therapy (Burvenich et al., 2016). A theranostic agent is one that is both diagnostic and therapeutic; for example, the agent can bind to DR5-expressing cells in COLO cell line xenografts and is a novel PET imaging reagent in human bioimaging (Burvenich et al., 2016). Therefore, it can monitor both the response to therapies and doses required (Burvenich et al., 2016) (Figure 2).

## Combination therapy using IAP antagonists

IAP antagonists can inhibit the caspase-inhibitory function of XIAP and also the cellular IAPs, cIAP1, and cIAP2, thereby enhancing apoptosis (Fulda, 2008). In solid tumor xenograft models using, for example, the breast cancer cell lines MDA-MB-231 and 2LMP TNBC, anti-tumor activity was demonstrated when the DR5 monoclonal antibody, CTB-006, was used together with the IAP antagonist APG-1387 (Li et al., 2021). Phase 1 dose-escalation studies have demonstrated the tolerability of IAP antagonists in the treatment of advanced solid tumors, such as breast cancer and lymphoma, in combination with pembrolizumab (Rasco et al., 2020). Phase I/II clinical trials for head and neck cancers have shown promising results (Kansal et al., 2023). Strong anti-tumor activity has been demonstrated with the combination of anti-DR5 agonist IGM-8444 with the SMAC mimetic, birinapant, in preclinical models using a range of cell lines and PDX models, including sarcoma; for example, 7/9 animals were tumor-free in a model using the HT1080 cell line (Wang et al., 2022), which has also been used to model dedifferentiated chondrosarcoma (Pringle et al., 2022). The same combination of anti-DR5 agonist IGM-8444 with birinapant is taking place for relapsed, refractory, or newly diagnosed cancers, which include soft tissue sarcoma and chondrosarcoma, in a phase 1a/1b study (ClinicalTrials.gov identifier: NCT04553692) and is still recruiting.

## Stem cells expressing TRAIL for cancer treatment

The use of stem cells that can migrate to tumors and deliver therapeutic agents has expanded the options available for cancer therapy and skeletal tissue regeneration (Fakiruddin et al., 2018; Gamie et al., 2012; Kamalabadi-Farahani et al., 2018). They can also be genetically engineered to overexpress the TRAIL ligand (Stamatopoulos et al., 2019). The metastatic tumor burden has been found to decrease with the administration of human mesenchymal stem cells (MSCs) expressing TRAIL in a pulmonary metastasis model (Loebinger et al., 2009). Since these early findings, developments have been made in terms of how stem cells can be used to express TRAIL to induce apoptosis in cancer. Human bone marrow-derived MSCs (BMMSCs) have been used previously to deliver membrane-bound TRAIL (either genetically or via TNF-α pre-activation) to breast cancer cells in an animal model and successfully induce apoptosis and reduce the tumor signal. The use of BMMSCs as a mode of delivery for therapeutic agents has been decreasing (Patrick et al., 2020), and other stem cell types have been utilized, such as those easier to obtain, such as umbilical cord-derived stem cells and human placenta-derived MSCs (Aldoghachi et al., 2023). More recently, human placenta-derived MSCs with curcumin-loaded chitosan particles have been utilized as a tumor-tropic therapy (Xu et al., 2019). Curcumin is reported to be a chemopreventive agent for oral squamous cell cancer (Maulina et al., 2019). MSCs expressing TRAIL have been studied *in vitro* and in mouse models of breast cancer with promising effects (Loebinger et al., 2009). MSCs have the ability to reside or engraft preferentially in tumors and their micrometastases and are an ideal candidate to deliver antineoplastic agents to the tumor site (Loebinger et al., 2009). Another agent investigated that upregulates DR5 is chrysanthemulide A (CA), which has been shown to induce apoptosis in osteosarcoma cells via JNK-mediated autophagosome accumulation (Zhuo et al., 2019).

MSCs can kill cancer cells *in vitro* and *in vivo,* and with sensitizing agents such as small-molecule inhibitors, TRAIL alone, or delivered with MSCs, the vehicle sensitizing agent can be enhanced (Fakiruddin et al., 2018). Stem cells engineered to express TRAIL can kill glioblastoma cells or hepatocellular carcinoma cells *in vitro*. Studies are ongoing utilizing this approach for the treatment of lung cancer (Patrick et al., 2020). The lung delivery of these umbilical cord-derived MSCs can be visualized using PET-CT in a novel study that uses 89Zr-oxine labeling to help evaluate the biodistribution of the MSCs and the amount migrating to the lungs (Patrick et al., 2020).

An ongoing trial known as TACTICAL is a multi-centered, randomized, double-blinded trial assessing the efficacy of MSC-TRAIL for lung adenocarcinoma, which combines the first-line standard-of-care chemotherapeutic agents, including pembrolizumab, with MSC-TRAIL, generated by transducing MSCs with a lentiviral vector to express TRAIL (ClinicalTrials.gov Identifier: NCT03298763) (Davies et al., 2019). It contains a first-in-man ^89^Zr-oxine cell-labeling arm to help obtain a better understanding of cell-based therapies. It is currently still recruiting patients, with an estimated study completion time of 1 September 2025 (Davies et al., 2019). If the trial is successful, it will add further confidence to the use of allogenic stem cell therapy for cancer.

Further modifications of TRAIL delivery and the use of stem cells to enhance the efficacy of MSC-TRAIL have involved modifications to target TRAIL delivery to cancer cells expressing specific antigens as part of a bi-functional strategy. TRAIL has been combined with a truncated anti-GD2 chimeric antigen receptor (GD2 tCAR) and delivered by MSCs in a novel strategy against metastatic Ewing’s sarcoma, demonstrating affinity and killing of lesions in the lung but not in the liver (Golinelli et al., 2022).

Research also continues into the pre-activation of stem cells using TNF-α to stimulate the expression of TRAIL in a bioactive membrane-bound form on the surface of the cell. Recently, it has been shown that dental tissues such as the periodontal ligament contain stem cells that have the main properties of MSCs, which can be expanded *ex vivo* (Di et al., 2023) and were found to express TRAIL at the protein and RNA levels when pre-activated using TNF-α. The stem cells migrated toward cancer cells when co-cultured with primary head and neck squamous cell carcinoma cells and induced apoptosis (Di et al., 2023). Further studies will require the comparison of different stem cell sources for TNF-α pre-activation and induction of apoptosis, biodistribution, and appropriate delivery of sufficient amounts to tumor sites (Ohara et al., 2024).

## Conclusions and future directions

Targeting DR5 remains a promising approach to help induce cell death by apoptosis or necroptosis (in combination with agents such as the SMAC mimetic birinapant) in the cancer cells of difficult-to-treat malignancies such as sarcoma. TRAIL therapeutics have had limited therapeutic efficacy and progress to phase 2 trials; however, the development of novel drugs and combination therapies allows for progress and advancements to phase 3 trials (Schneider et al., 2010). The remaining challenges include identifying patients with high levels of expression of DR5 and further study in 3D models, such as organoids, where resistant cells may limit the killing capabilities of the agent. Further investigation into the development of PDX mouse models that can help replicate tumor growth, local invasion, and metastatic potential, is needed. Ongoing phase 2 trials will demonstrate whether the strategies used in promising *in vivo* studies, utilizing antibodies that have superseded the early agonistic antibodies that had a weak ability to induce receptor multimerization, can be replicated in patients. Advancements in nanotechnology and nanoparticle-mediated TRAIL delivery, as well as MSC-mediated TRAIL delivery, have also proved to be successful *in vitro* and in preclinical animal models (Sadeghnezhad et al., 2019; Gampa et al., 2023; Aldoghachi et al., 2023). The progress in imaging strategies for the detection of DR5 levels and the potential to combine this with enhanced triggering of DR5 activity warrant further investigation as a strategy with standard-of-care chemotherapeutic agents, in particular in the difficult-to-treat malignancies, to enhance cytotoxicity and improve survival.
